# Factor investing and asset allocation strategies: a comparison of factor versus sector optimization

**DOI:** 10.1057/s41260-021-00225-1

**Published:** 2021-05-29

**Authors:** Wolfgang Bessler, Georgi Taushanov, Dominik Wolff

**Affiliations:** 1 Deutsche Börse Senior Professor of Empirical Capital Market Research, University of Hamburg, Moorweidenstrase 18, 20148 Hamburg, Germany; 2 Gothaer Asset Management GmbH, Cologne, Germany; 3 Technical University Darmstadt and Deka Investment GmbH, Frankfurt, Germany

**Keywords:** Asset allocation, Portfolio optimization, Factor investing, Factor versus sector allocation, G17, G11, C53

## Abstract

Given the tremendous growth of factor allocation strategies in active and passive fund management, we investigate whether factor or sector asset allocation strategies provide investors with a superior performance. Our focus is on comparing factor versus sector allocations as some recent empirical evidence indicates the dominance of sector over country portfolios. We analyze the performance and performance differences of sector and factor portfolios for various weighting and portfolio optimization approaches, including “equal-weighting” (1/N), “risk parity,” minimum-variance, mean-variance, Bayes–Stein and Black–Litterman. We employ a sample-based approach in which the sample moments are the input parameters for the allocation model. For the period from May 2007 to November 2020, our results clearly reveal that, over longer investment horizons, factor portfolios provide relative superior performances. For shorter periods, however, we observe time-varying and alternating performance dominances as the relative advantage of one over the other strategy depends on the economic cycle. One important insight is that during “normal” times factor portfolios clearly dominate sector portfolios, whereas during crisis periods sector portfolios are superior offering better diversification opportunities.

**Supplementary Information:**

The online version contains supplementary material available at 10.1057/s41260-021-00225-1.

## Introduction

For many decades, favored asset allocation strategies focused on country or sector portfolios. However, more recently factor investing emerged as a popular new approach. The idea of factor investing is to diversify a portfolio among the underlying characteristic risk factors, introduced in the literature for explaining stock returns. The main objective of this research is to investigate whether factor-based portfolios perform superiorly relative to sector-based portfolios. Moreover, we are interested in the question whether factor timing adds value and examine whether dynamic factor investment strategies outperform a static multifactor benchmark. We extent the earlier research of Briere and Szafarz (2021) by focusing on investable factor and sector indices and by analyzing a variety of different out-of-sample investment strategies.

Extending the single-factor Capital Asset Pricing Model (Sharpe 1964) to multifactor models has a long tradition. Following a decade of research in arbitrage pricing theories (APT), Chen et al. (1986) introduced a multifactor model employing pre-specified macroeconomic variables. Fama and French (1993) extended and shifted the multifactor analysis toward characteristic factors, dividing the systematic risk spectrum initially into three core components. This offered researchers and investors the opportunity to better differentiate between the various sources of risk and risk premium in capital markets. Carhart (1997) extended this to a four-factor model by including a momentum factor, and Fama and French (2015, 2018) added two additional factors to their own model resulting in a five-factor model or six-factor model when adding the momentum factor. Other often-employed factors are “dividends” (Fama and French 1988, 1993), “liquidity” (Pástor and Stambaugh 2003), “betting-against-beta” (Frazzini and Pedersen 2014) and “quality-minus-junk” (Asness, et al. 2017).

The inroad of factors as asset classes into asset management, however, occurred through a government-mandated report for the Norwegian government pension fund conducted by Ang et al. (2010). The analysis suggested that systematic risk factors could explain a substantial part of the returns of actively managed funds. Consequently, asset management should employ factors not only to measure fund performance but also to construct portfolios. This research suggested that the asset allocation strategies should shift from the classical diversification across sectors or countries toward a factor-based approach. Subsequently, factor allocation strategies experienced a rising popularity among portfolio managers (Amenc et al. 2016).

In this study, we analyze different asset allocation models and compare their portfolio performance. As assets, we employ factor and sector indices, which are investable at low cost via exchange-traded funds (ETFs). We compare sector and factor allocations rather than comparing country and factor allocations as Bessler et al. (2021) provided recent evidence that sectors dominate country allocations. We measure the portfolio performance of both strategies first by analyzing the risk and return profiles and Sharpe ratios, and second, by comparing the alphas based on multifactor regressions. We also analyze whether factor-based optimized portfolios generate higher returns and higher multifactor alphas compared to a buy-and-hold factor investment. Third, we split the full period into sub-periods and compare the time variation of the sector and factor performance for each period. For longer investment horizons, we find that factor are superior to sector allocations. For shorter periods, the results are less consistent.

The rest of this research we structure as follows. In Sect. 2, we review the literature and outline our general ideas. The optimization methodology is detailed in Sect. 3, and the data are given in Sect. 4. In Sect. 5, we present the empirical results of the optimization processes as well as some further analyses. Chapter 6 concludes.

## Literature review

Asness et al. (2013) were among the first to document the diversification potential of factor-based portfolios.^1^ They studied the relationship between value and momentum characteristics and find strong, predominantly negative correlations between the underlying factors. Therefore, portfolios exposed to value or momentum characteristics should offer diversification benefits, substantially reducing risk. Both studies further report considerable value and momentum premium across different asset classes and markets, suggesting that a factor-based asset allocation approach provides higher diversification, return and performance potential relative to the “classic” strategies such as country or sector allocations.

Bender et al. (2010) extend the ideas of Asness et al. (2013) by increasing the number of factors in the portfolio allocation process. Their findings support the ideas that combining different factors in an investment strategy results in a superior performance relative to traditional asset allocation approaches. While an equally weighted portfolio of different factors and a traditional equity–bond portfolio yield similar average returns, factor portfolios contain much lower risk (less than one third of the risk of the traditional equity–bond portfolio), hence offering a higher performance (Sharpe ratio). In a similar study, Blitz (2011) uses the long components of four different factors (size, value, momentum and low volatility) as portfolio constituents. When extending the equally weighted optimized portfolios by including return predictions, the results confirm that factor-based allocation strategies provide significantly higher returns compared to a classical allocation strategy.

Hjalmarsson (2011) also extents the approach of Asness et al. (2013) by adding additional factors to the allocation process. Equal-weighted portfolios of seven or more factors generate higher Sharpe Ratios than any of the observed single-factor portfolios or the Asness et al. (2013) two-factor portfolios. This indicates that the classical diversification effect (decreasing risk when increasing the number of assets) might also apply to factor portfolios. In another study, Melas et al. (2011) simulated different fund strategies and organizational structures. Measuring the performance improvements of factor-based portfolios, the authors conclude that the focus of institutional investment funds should shift away from active asset allocations toward allocation between different sources of systematic risk such as factors. Consequently, the allocation of large portfolios should predominantly focus on beta, with a smaller attention to alpha management, which is in accordance with the ideas of Ang et al. (2010).

In fact, Ang (2010) describes the interaction between factors and returns as fundamental and equivalent to the relationship between “nutrients and food.” Following this concept, Ang and Kjaer (2012) argue that professional portfolio management should organize their allocation strategies based on the beta (“the nutrients”) instead of seeking the highest alpha. Consequently, the best strategy contains a diversification approach across factors, because “factors go beyond assets.”^2^ Ilmanen and Kizer (2012) extend this view of Ang and Kjaer (2012) by comparing the effectiveness of “asset class” and “dynamic factor” diversifications. They report that factor-based portfolios contain much lower overall correlations than sector-based portfolios. Their performance comparison of equally weighted factor and “traditional asset” portfolios supports this view with factor portfolios yielding noticeably larger Sharpe ratios. However, the analysis of Ilmanen and Kizer (2012) is limited only to equally weighted portfolios and only for the period up to 2010. We extent the literature by comparing sector and factor portfolios for various dynamic asset allocation strategies and different portfolio optimization techniques up to the end of 2020.

As the factor portfolios’ popularity increased, Kahn and Lemmon (2016) predict substantial changes in active portfolio management. They believe that the smart beta and factor investment might replace a large part of the typical alpha-focused active management. Briere and Szafarz (2021) compare factor- and sector-based allocations and conclude that sector investing helps to reduce risks during crisis periods, while factor investing can boost returns during expansion periods. Dichtl et al. (2020) provide further evidence supporting the diversification effects of factors by developing a “factor completion” framework by incorporating the benefits of factors in a standardized allocation process.

The analysis of Briere and Szafarz (2021) builds on Fama–French factors, which are not directly investable. However, implementing portfolio strategies based on these factors involves large portfolio turnover and transaction costs, because not only Fama–French factor portfolios require updating on a monthly basis but also factor portfolio weights need adjusting over time. Therefore, we analyze factor versus sector strategies from a practical perspective and build our analysis on factor and sector indices, which are investable at low cost via ETFs. We also explicitly account for transactions costs. Moreover, relative to Briere and Szafarz (2021), we test a variety of out-of-sample allocation strategies to analyze the benefits of factor versus sector investing for different investor types and investment styles.

The growing academic interest in factor returns and asset allocation strategies, however, also resulted in an opposite and critical perspective. Arnott et al. (2016) distinguish between structural and situational sources of returns where the structural “value adds”—in contrast to the situational—are stable in time and therefore a reliable source of future returns.^3^ The authors argue that in the recent years the prices of factor portfolios have grown too high due to the excessive demand by performance seeking portfolio managers. Consequently, the probability of generating persistently high returns in the future has declined, which also cause the situational character of the factor returns. Dimson et al. (2017) point out that the core meaning of factors lies in the co-movement of the group of stocks sharing specific attributes. They interpret the counter-movement in some of the observed groups as an essential driver for portfolio risk reduction. The authors, however, do not find evidence for a sustainable return premium in the observed factors.

A critical question is whether factor timing or factor tilting add value compared to a static factor allocation. In general, “factor tilting” describes the relative over- or underweighting of a factor compared to other factors. The benefit of factor timing critically depends on the predictability of factor returns. Dichtl et al. (2019) investigate the benefits of parametric portfolio strategies for factor timing and tilting, indicating that this might significantly improve the factor portfolio performance. However, benefits in the practical portfolio management deteriorate after including transaction costs.

## Methodology

In this section, we present a variety of asset allocation models and out-of-sample estimation procedures that we implement in our study. We follow the results of DeMiguel et al. (2009) that no portfolio construction technique is persistently superior to 1/N and use this approach as our benchmark. We then employ a variety of weighting and optimization techniques, analyze their relative performance and evaluate whether they outperform the 1/N strategy. Our assets are sector and factor indices, and therefore, our research objective is to compare the benefits of various factor- and sector-based allocation strategies. These strategies include the naive “equally weighted” (1/N) portfolio as benchmark, the risk-based asset allocation rules “risk parity” (RP) as well as four portfolio optimization approaches: minimum variance (MinVar), mean–variance (MV), Bayes–Stein (BS) and Black–Litterman (BL).

We structure our optimization process as follows: The weights for each portfolio are determined at the last trading day of each month, based on all previous information. We calculate the portfolio returns for the following month by multiplying the calculated weights with the component returns of the corresponding month. We include 20 basis points of transaction costs, which is reasonable, given that we implement the sector and factor allocations with exchange-traded funds (ETFs). We repeat this process monthly by moving the sample period one month forward and by computing the optimized weights for the next month. Taking the forecast and optimization calibration periods into account, we compare all asset allocation strategies for these two asset classes, sectors and factors, for the out-of-sample period from May 2007 to November 2020.

A critical aspect in optimal portfolio allocation decisions besides the optimization model is the input data, for which we use historical average returns. As a robustness check, we employ different window lengths ranging from 12 to 60 months. We also employ the cumulated average (CA) strategy, which includes all observations from the beginning of the sample period up to the current observation. Further, to test the robustness of our results, we use different investment constraints such as long-only portfolios and portfolios with short positions. We also investigate the effects of constraints on performance by relaxing the constraints in the range between −35% (50%) (short positions) and +35% (50%) (maximum weight of a constituent in our portfolio). Next, we discuss the different asset allocation approaches for our investment strategies.

### Asset allocation models

Both academia and the asset management industry analyzed and implemented a large variety of different asset allocation approaches. These models range from naive to sophisticated models. Our description begins with the rather simple rule-based weighting methods such as “equal-weighting” or “risk parity” and continues in the next section with the optimization-based methods: minimum variance, the classical mean–variance, the Bayes–Stein, as well as the Black–Litterman optimization approaches.

#### Naive diversification 1/N

Being one of the most prominent allocation methods among private investors according to Benartzi and Thaler (2001), we implement the 1/N wealth distribution approach, which allocates equal weights to every asset. Therefore, given the number of constituents available for portfolio construction (6 factor and 10 sector portfolios), 1/N always allocates 16.67% and 10% of the portfolio to every factor or sector index, respectively. As for all other allocation strategies, we rebalance the portfolios monthly.

#### Risk parity

The risk parity (RP) approach is another prominent weighting method, widely adopted by both academia and professional portfolio management. With the growing interest in the so-called smart beta strategies, a large number of mutual funds, index providers, pension funds, endowments and other long-term investors have adopted this strategy.^4^ The basic idea of the risk parity weighting is that each portfolio component contributes equally to portfolio risk, neglecting correlations between asset returns. Therefore, assets are weighted counter-proportional to their sample variance ${{\widehat{\sigma }}_{i}}^{2}$.

1 $${\omega }_{i}=\frac{1/{\widehat{\sigma }}_{i}^{2}}{{\sum }_{i=1}^{N}\left(1/{\widehat{\sigma }}_{i}^{2}\right)}$$

Anderson et al. (2012) report that risk parity strategies perform well and usually even outperform 1/N, value-weighted or 60/40 portfolios. The risk parity approach exploits the low-volatility anomaly, according to which low-volatility assets usually earn a higher premium per unit of volatility than high-volatility assets (Baker et al. 2011; Frazzini and Pedersen 2014). It therefore underweights high-volatility assets and overweight low-volatility assets. We next turn from the simple to the optimization-based asset allocation approaches.

#### Minimum variance

A simple and robust optimization-based strategy is the minimum variance approach (MinVar), which is increasingly popular among institutional investors such as quantitative mutual funds or exchange-traded funds.^5^ The objective of the MinVar strategy is to minimize portfolio volatility. The minimization problem is:

2 $$\underset{\omega }{\mathrm{min}}{\omega }^{{\prime}}\Sigma \omega$$

where *ω* is the vector of portfolio weights and *Σ* is the covariance matrix. The main advantage of the minimum variance approach is that it does not require any return estimates, which are highly vulnerable to estimation errors and therefore the main source of sub-optimal allocation decisions. As risk parity, this concept utilizes the observation that low-risk assets often generate a higher return premium per unit of volatility (low-volatility anomaly). The core difference between the MinVar and the RP strategy is, however, that the MinVar strategy is a portfolio optimization-based approach that also takes the correlation of asset returns into account.

#### Mean–variance

Mean–variance (MV)—in contrast to minimum variance—also includes return predictions for optimizing the risk–return trade-off (Markowitz 1952). The mean–variance optimization problem is:

3 $$\underset{\omega }{\mathrm{max}}U={\omega }^{{\prime}}\mu -\frac{\delta }{2}{\omega }^{{\prime}}\Sigma \omega$$

where U is the investor’s utility, *μ* is the vector of expected return estimates and *δ* is the risk aversion coefficient.^6^ To implement the mean–variance strategy, we use the sample mean $\widehat{\mu }$ and the sample covariance matrix $\widehat{\Sigma }$ as described above. The optimization is subject to two restrictions. First, we include a budget restriction, ensuring that portfolio weights sum to one and second, we prohibit short positions in our base case. The risk aversion coefficient is set to five (for a discussion of parameter settings see Bessler et al. 2017).

#### Bayes–Stein

Developed by Jorion (1985), the Bayes–Stein (BS) model extends the MV strategy attempting to reduce estimation errors in the return and volatility forecasts by relying on a Bayesian estimation approach (Stein 1956; James and Stein 1956). The optimization procedure itself is identical as in the MV approach presented in equation (3).

The sample means as expected returns $\widehat{\mu }$ are shrunk toward the expected returns of the minimum variance portfolio ${\widehat{\mu }}^{min}$ given the shrinkage factor $\widehat{\gamma }:$

4 $$\hat{\mu }_{{\min }} = \hat{w}_{{\min }}^{'} ~\hat{\mu } = \frac{{\vec{1}^{'} \hat{\Sigma }^{{ - 1}} }}{{\vec{1}^{'} \hat{\Sigma }^{{ - 1}} \overset{\lower0.5em\hbox{$\smash{\scriptscriptstyle\rightharpoonup}$}} {1} }},$$

5 $${\widehat{\mu }}_{BS}=\left(1-\widehat{\gamma }\right)\widehat{\mu }+\widehat{\gamma }{\widehat{\mu }}_{min}\overrightarrow{1} \quad \mathrm{ with}\quad \widehat{\gamma }=\frac{N+2}{\left(N+2\right)+T{\left(\widehat{\mu }-{\widehat{\mu }}^{min}\overrightarrow{1}\right)}^{{\prime}}{\Sigma }^{-1}\left(\widehat{\mu }-{\widehat{\mu }}^{min}\overrightarrow{1}\right)}$$

The covariance matrix, is also adjusted following the Jorion (1986) methodology and calculated as follows:

6 $${\widehat{\Sigma }}_{BS}=\frac{T-1}{T-N-2}\widehat{\Sigma } .$$

where $\overrightarrow{1}$ is a vector of ones, $\widehat{\Sigma }$ denotes the unbiased sample covariance matrix, N the number of assets and T is the number of observations (Jorion 1986). Subsequently, we apply the same optimization procedure, constraints and estimation windows as for the MV approach.

#### Black–Litterman

Another approach to mitigate the noise problems in portfolio optimization was proposed by Black and Litterman (1992). Similarly to the MV and BS, the Black–Litterman (BL) model is popular among quantitative asset managers (Satchell and Scowcroft 2000; Jones et al. 2007). BL combines “implied” returns calculated from a benchmark portfolio via reverse optimization with explicit return forecasts or “views” taking the correlation structure between the assets into account (Black and Litterman 1992; Lee 2000). The major advantage of the BL model is that it integrates the reliability of the forecasts into the allocation process. The vector of the return forecasts according to Black and Litterman (1992) is estimated as follows:

7 $${\widehat{\mu }}_{BL}={\left[{\left(\tau\Sigma \right)}^{-1}+{P}^{{\prime}}{\Omega }^{-1}P\right]}^{-1}\left[{\left(\tau\Sigma \right)}^{-1}\Pi +{P}^{{\prime}}{\Omega }^{-1}Q\right]$$

where *П* is the vector of “implied” returns, *Σ* is the covariance matrix and *Q* is the vector of the investor’s return estimates. The reliability of the return predictions is contained in the diagonal matrix *Ω* and can be measured as the variance of the realized prediction errors in the estimation window*. P* denotes an identity matrix and *τ* a scalar that calibrates the tracking error to the benchmark portfolio.^7^ The BL model also adjusts the covariance matrix. Following Satchell and Scowcroft (2000), the covariance matrix is computed as:

8 $${\Sigma }_{BL}=\Sigma +{\left[{\left(\tau\Sigma \right)}^{-1}+{P}^{{\prime}}{\Omega }^{-1}P\right]}^{-1}$$

After computing expected returns (equation 7) and the covariance matrix (equation 8), the portfolio weights are calculated in the same fashion as for the MV and the BS methods—via traditional risk–return optimization, maximizing the investor’s utility as presented in equation (3). The rest of the relevant parameters such as risk aversion coefficient, optimization procedure and constraints remain the same across all risk–return strategies. Following Bessler and Wolff (2015), we implement a sample-based version of the BL model.^8^

### Performance measures

To evaluate the performance of factor- versus sector-based portfolios, we employ several performance measures. We compute the portfolio’s average out-of-sample return and volatility as well as the out-of-sample Sharpe ratio. Following Opdyke (2006), we test whether the difference in Sharpe ratios of two portfolios is significant. This test is applicable under very general conditions—stationary and ergodic returns. Most importantly for our analysis, the test permits autocorrelation and non-normal distribution of returns and allows for a likely high correlation between the portfolio returns. To provide further evidence and to make our study better comparable to the recent literature, we implement different factor models for computing multifactor alphas as risk-adjusted performance measures. The first factor model contains the six Fama–French (2018) long–short factors from Kenneth French’s website: “Market,” Value (“HML”), Size (“SMB”), Quality (“RMW”), Investment (“CMA”) and Momentum (“MOM”) augmented by the betting-against-beta factor (“BAB”) proposed by Frazzini and Pedersen (2014). This analysis allows us to determine whether the performance of our factor or sector portfolios is fully attributable to the known risk factors or whether they provide significant multifactor alphas.

The second factor model we employ to compute multifactor alphas includes the same six MSCI factors, which we employ as asset classes in the factor portfolios. This analysis allows us to investigate whether dynamically adjusting factor allocations adds value over a buy-and-hold factor portfolio. Finally, we compare the periodical performance of the two strategies depending on the state of the economy.

## Data

Our two investment strategies include 10 sector and 6 factor indices, which are investable with ETFs. Both sector- and factor-based portfolios contain only US stocks. The 16 total return indices are downloaded from Bloomberg on a monthly basis for the longest jointly available period from January 1999 to November 2020. The dataset includes 262 monthly return observations for each index. For implementing the portfolio optimization strategies, we need up to five years (60 months) for estimating the optimization inputs (average returns and the covariance matrix). Three more years of data are required in the Black–Litterman optimization for determining the reliability of return estimates. Therefore, our out-of-sample evaluation period ranges from 2007 to 2020 and includes 166 months.

The sector indices, as presented in Table 1 Panel A, are based on the constituents of the S&P 500 index. The indices (Factset) include the following sectors: “Industrials,” “Consumer Discretionary,” “Utilities,” “Energy,” “Consumer Staples,” “Information Technology,” “Communication Services,” “Health Care,” “Financials,” “Materials.”

**Table 1:** Description of sector and factor indices

Panel A: Description of sector indices	
Sector	Index
1. Industrials	S&P 500/Industrials—USD Total Return Index
2. Consumer Discretionary	S&P 500/Consumer Discretionary—USD Total Return Index
3. Utilities	S&P 500/Utilities—USD Total Return Index
4. Energy	S&P 500/Energy—USD Total Return Index
5. Consumer Staples	S&P 500/Consumer Staples—USD Total Return Index
6. Information Technology	S&P 500/Information Technology—USD Total Return Index
7. Communication Serv.	S&P 500/Communication Services—USD Total Return Index
8. Health Care	S&P 500/Health Care—USD Total Return Index
9. Financials	S&P 500/Financials—USD Total Return Index
10. Materials	S&P 500/Materials—USD Total Return Index

Panel B: Description of factor indices	
Factor	Index
1. Momentum	MSCI USA Momentum USD Total Return Index
2. MinVol	MSCI USA Minimum Volatility Total Return Index
3. Value	MSCI USA Value Total Return USD Index
4. Quality	MSCI USA Quality Total Return USD Index
5. Size	MSCI USA Equal Weighted (Size) Total Return USD Index
6. Carry	MSCI USA High Dividend Yield Total Return Risk Premia Index

From the large number of factors discussed in the literature, only a few factors are investable at low cost, for instance, through ETFs. Our factor portfolios, as presented in Table 1 Panel B, consist of the following 6 investable MSCI US long-only factor indices with a sufficient data history. The momentum factor (Carhart 1997), the size factor (Fama and French 1993), the value factor (Fama and French 1993), the quality factor (Fama and French 1993), the carry factor (based on “dividend yield” as proposed by Fama and French 1988, 1993) and the low volatility/low beta factor (Frazzini and Pedersen 2014).

Tables 2 and 3 display the statistical properties of the sector and factor indices, respectively. In general, the mean returns for the factor and the sector indices are similar. For the factor indices, the returns range from 0.45% (“Value”) to 0.77% per month (“Momentum”). For the sector indices, the minimum and the maximum values are, 0.19% for the “Consumer Services” sector and 0.72% for the “Consumer Discretionary” per month. The standard deviations of returns reveal that, on average, the factor indices have lower risk with 4.42% per month compared to sector indices with 5.50% per month. Comparing the minimum and maximum values between both index groups, we observe the lowest risk for the “Consumer Staples” sector (3.46%) compared to the low volatility factor (3.79%). We measure the highest risk for the factor “Size” with 5.1%, whereas the highest risk for the sector group is 7.25% (“IT”).

**Table 2:** Summary statistics and correlation matrix of the sector indices

	Industrials	Cons. Disc.	Utilities	Energy	Cons. Staples	IT	Com. Srvs	Health Care	Financials	Materials
	*Panel A: Summary statistics*									
Mean	0.62%	0.72%	0.59%	0.39%	0.61%	0.62%	0.19%	0.62%	0.32%	0.67%
Median	1.08%	0.86%	1.16%	0.76%	0.89%	1.48%	0.78%	1.03%	0.91%	0.86%
Max	16.43%	18.69%	12.83%	26.07%	11.09%	20.13%	28.48%	11.91%	20.18%	21.57%
Min	− 21.30%	− 21.31%	− 15.06%	− 42.76%	− 11.56%	− 32.85%	− 16.87%	− 13.35%	− 30.53%	− 24.94%
Std. Dev.	5.42%	5.36%	4.59%	6.84%	3.46%	7.25%	5.71%	4.14%	6.45%	6.10%
Skewness	− 0.73	− 0.34	− 0.72	− 0.84	− 0.65	− 0.79	− 0.04	− 0.50	− 0.92	− 0.32
Kurtosis	5.31	4.42	3.94	9.53	4.18	5.20	5.08	3.49	6.86	4.65
Jarque-Bera	81.51	27.05	32.43	496.78	33.42	80.15	47.45	13.68	199.69	34.11
(p-Value)	0.10%	0.10%	0.10%	0.10%	0.10%	0.10%	0.10%	0.64%	0.10%	0.10%
Obs	262	262	262	262	262	262	262	262	262	262
	*Panel B: Correlation matrix*									
Industrials	1.00									
Cons. Disc.	0.85	1.00								
Utilities	0.39	0.30	1.00							
Energy	0.65	0.55	0.40	1.00						
Cons. Staples	0.57	0.54	0.48	0.41	1.00					
IT	0.66	0.74	0.20	0.41	0.28	1.00				
Com. Srvs	0.54	0.59	0.32	0.42	0.43	0.54	1.00			
Health Care	0.57	0.56	0.38	0.41	0.66	0.36	0.42	1.00		
Financials	0.82	0.77	0.36	0.56	0.57	0.52	0.46	0.59	1.00	
Materials	0.84	0.78	0.34	0.68	0.52	0.58	0.46	0.49	0.72	1.00

This table provides summary statistics and the correlation matrix for sector returns for the full dataset ranging from January 1999 to November 2020

**Table 3:** Summary statistics and correlation matrix of the factor indices

	Momentum	MinVol	Value	Quality	Size	Carry
	*Panel A: Summary statistics*					
Mean	0.77%	0.61%	0.45%	0.64%	0.68%	0.57%
Median	1.19%	0.90%	0.63%	0.89%	0.86%	0.84%
Max	13.64%	10.08%	10.94%	11.42%	16.35%	12.5%
Min	− 14.12%	− 12.39%	− 16.76%	− 13.37%	− 20.22%	− 13.6%
Std. Dev.	4.60%	3.79%	4.55%	4.31%	5.10%	4.20%
Skewness	− 0.38	− 0.55	− 0.72	− 0.52	− 0.60	− 0.58
Kurtosis	3.38	3.70	4.22	3.37	4.59	4.36
Jarque-Bera	7.95	18.45	38.68	13.11	43.19	35.00
(p-Value)	2.43%	0.28%	0.10%	0.72%	0.10%	0.10%
Obs	262	262	262	262	262	262
	*Panel B: Correlation matrix*					
Momentum	1.00					
MinVol	0.81	1.00				
Value	0.77	0.92	1.00			
Quality	0.86	0.89	0.89	1.00		
Size	0.81	0.88	0.95	0.89	1.00	
Carry	0.67	0.93	0.93	0.81	0.84	1.00

This table provides summary statistics and the correlation matrix for factor returns for the full dataset ranging from January 1999 to November 2020

One of the most important statistical aspects directly affecting the risk and therefore the performance of an optimized portfolio is the correlation structure among the assets. In general, the group of assets with the lower correlation offers better diversification opportunities, which, however, does not necessarily mean higher performance. For the sector indices, the highest correlated pairs are the following: “Industrials”—“Consumer Discretionary” (0.85), “Industrials”—“Materials” (0.84), as well as “Industrials”—“Financials” (0.82). The pairs with the lowest correlation in the sector group are “Utilities”—“IT” (0.20), “Consumer Staples”—“IT” (0.27) as well as “Utilities”—“Consumer Discretionary” (0.30).

Both, maximal and minimal correlation coefficients in the factor indices are higher than for the sector group. The highest correlations we observe for factors are for the following pairs: “Quality”—“Value” (0.95), as well as between “Carry”—“MinVol” and “Carry”—“Value” (both 0.93). The lowest correlations in the factor group are the following: “Carry”—“Momentum” (0.67) followed by “Value”—“Momentum” (0.77).

With respect to the optimization process, our insights from the descriptive statistical analysis are twofold. First, the correlations between factor indices are noticeably higher than between sectors, offering lower diversification opportunities. Second, average monthly returns were slightly higher for factor indices than for sectors, indicating higher portfolio returns. Therefore, it is essential to employ the different portfolio optimization algorithms to provide empirical evidence whether sectors or factors provide the better trade-off between risk and return and therefore are the superior building blocks for an optimal investment strategy.

## Empirical results

We structure this section as follows. Section 5.1 reports the portfolio performance for the full period, Section 5.2 contains additional risk analyses and Section 5.3 shows the sub-period results.

### Portfolio performance full period

In this section, we report the full period results by providing the following performance results: (a) full period analysis of Sharpe ratios, (b) sensitivity analysis of changing constraints, (c) risk and return analysis, (d) multifactor performance analysis and (e) multifactor alpha differences between both strategies.

- Full period analysis of Sharpe ratios (Table 4).

**Table 4:** Sharpe ratios

Optimization method	Estimation window	Weight constraints					
		[0%; +35%]		[−35%; +35%]		[−50%; +50%]	
		(1)	(2)	(3)	(4)	(5)	(6)
		FI	SI	FI	SI	FI	SI
1/N		1.17	0.90				
Risk parity		1.15	1.00				
MinimumVar.		1.15	1.02	1.15	1.06	1.07	1.05
Black–Litterman	CA	1.15	0.96	1.14	0.96	1.14	0.98
	12	1.19	0.87	1.17	0.73	1.20*	0.67
	36	1.18	0.92	1.18	0.87	1.20	0.90
	60	1.16	1.01	1.17	0.98	1.18	1.00
Mean–variance	CA	1.19*	0.70	1.24*	0.64	1.17**	0.37
	12	1.22	0.81	1.21	0.82	1.22*	0.58
	36	1.21	0.92	1.19*	0.66	1.17**	0.46
	60	1.26	0.99	1.30	1.00	1.38*	0.77
Bayes–Stein	CA	1.17	0.93	1.14	0.90	1.12	0.78
	12	1.23	0.87	1.24	0.88	1.24**	0.54
	36	1.20	0.96	1.16	0.95	1.17	0.82
	60	1.19	1.03	1.21	1.13	1.23	1.01

This table provides the annualized Sharpe ratios of the factor and sector portfolios (FI, SI). The evaluation period covers the entire sample period from May 2007 to November 2020. As return forecasts for the optimization models, we apply different moving averages, the length of which is displayed in the second column of the table. *, ** and *** indicate the significance of the Sharpe ratio difference tests between both portfolio types at the 10%, 5% and 1% level, respectively. The stars are displayed on the side of the portfolio with the higher Sharpe ratio and report the significance of the hypothesis that the higher Sharpe ratio is significantly higher over its counterpart

In Table 4, we report and compare the pairwise Sharpe ratios of the sector- and factor-optimized portfolios. We analyze six different asset allocation frameworks, which consist of equal weighting (1/N), two risk-based allocations (RP, MinVar) and the three risk–return optimizations (BL, MV, BS) approaches. For the risk–return optimization approaches, we employ four different estimation window lengths as model inputs. The portfolio constraint for our base case is a 35% allocation to one single asset (index) and no short positions. The second framework allows for a 35% long or 35% short position in any single index, whereas in the third framework we extend both limits to 50% for long and 50% for short position in a single index, allowing maximum flexibility for risk exposure management. This optimization framework (weighting or optimization algorithm, return forecast and constraints) we apply to both factor- and sector-based portfolios.

The initial results presented in Table 4 reveal that for all reported pair results, which are the differences between sector and factor portfolios, the factor allocations generate higher Sharpe ratios. Due to the relatively short history of the indices, however, the Sharpe ratio differences are statistically significant only for some portfolio pairs (according to the Opdyke (2006) test). Still, the Sharpe ratio differences are economically relevant and the information that factor portfolios dominate sector portfolios in all analyzed cases is a very strong outcome in favor of factor allocations.

(b) Sensitivity analysis of changing constraints

We next focus on the effects of different weight constraints (columns (1) to (6) in Table 4, respectively). We observe for the long–short allocations (maximum short and long positions between −35% and +35%, and between −50% and +50%) that the performance differences between the two allocation strategies become larger relative to the long-only case. The factor performance is relatively stable for different optimization constraints, whereas the performance of the sector allocations tends to deteriorate with more relaxed optimization restrictions.

We observe the highest performance difference for the mean–variance algorithm with the most relaxed portfolio restrictions (−50% to +50%). In contrast, the lowest performance difference occurs for the minimum variance approach, although factor portfolios outperform sector allocations. This is a rather surprising result, given that the average correlations between sectors were lower than between factor indices. The performance difference for the minimum variance approach gets even smaller when we further relax the optimization restrictions. The analysis of the risk and return structure of the portfolios in the next section provides further insights for explaining these results.

(c) Risk and return analysis

Separating Sharpe ratios into their two basic components return and risk offers some additional insights into the source of the performance differences between sector and factor allocations. We present the annualized mean returns as well as the risk (standard deviation, volatility) of the optimized portfolios in Table 5 . The benefits of the factor allocation originate not only from larger returns (Table 5, Panel A) but also from lower portfolio volatility (Table 5, Panel B). In all cases, regardless of the allocation environment (optimization algorithm, window length for optimization inputs and weight constraints), the factor portfolios reveal lower or equal risk as the sector portfolios. These outcomes are in accordance with the observation in Bender, et al. (2010) that factor-based diversification lowers portfolio risk. There is only one exception, in which the sector allocation provides a marginal lower volatility: the minimum variance portfolio with narrow weight restrictions. For the properties of the optimization methods, we identify some interesting details for the factor allocations. Similar to the Sharpe ratios, the risk profile of the factor portfolios closely relates to the constraints. The highest differences in risk of all optimization–forecast combinations occurs in the case with the most relaxed weight constraints (−50% and 50%; column 5 and 6 in Table 5).

**Table 5:** Annualized portfolio returns and volatilities

Optimization method	Estimation window	Weight constraints								
		[0%;+20%]			[−20%;+20%]				[−50%;+50%]	
		(1) FI	(2) SI		(3) FI		(4) SI		(5) FI	(6) SI
*Panel A: Annualized portfolio returns*										
1/N		**0.15**	**0.13**							
Risk parity		**0.15**	**0.13**							
MinimumVar.		**0.14**	**0.12**		**0.14**		**0.13**		**0.13**	**0.13**
Black–Litterman	CA	**0.15**	**0.12**		**0.14**		**0.12**		**0.14**	**0.13**
	12	**0.15**	**0.12**		**0.15**		**0.10**		**0.15**	**0.10**
	36	**0.15**	**0.12**		**0.15**		**0.11**		**0.15**	**0.12**
	60	**0.15**	**0.13**		**0.15**		**0.13**		**0.15**	**0.13**
Mean–variance	CA	**0.15**	**0.09**		**0.16**		**0.10**		**0.15**	**0.07**
	12	**0.16**	**0.12**		**0.16**		**0.14**		**0.16**	**0.12**
	36	**0.15**	**0.12**		**0.14**		**0.10**		**0.14**	**0.09**
	60	**0.16**	**0.13**		**0.16**		**0.14**		**0.16**	**0.14**
Bayes–Stein	CA	**0.15**	**0.11**		**0.14**		**0.11**		**0.14**	**0.11**
	12	**0.16**	**0.12**		**0.16**		**0.13**		**0.16**	**0.10**
	36	**0.15**	**0.12**		**0.14**		**0.13**		**0.14**	**0.13**
	60	**0.15**	**0.12**		**0.14**		**0.14**		**0.14**	**0.14**
*Panel B: Annualized portfolio volatilities*										
1/N		0.13	0.14							
Risk parity		0.13	0.13							
MinimumVar.		0.12	0.11	**0.12**		**0.12**		**0.12**		**0.12**
Black–Litterman	CA	**0.12**	**0.12**	**0.12**		**0.12**		**0.12**		**0.12**
	12	**0.13**	**0.13**	**0.12**		**0.13**		**0.12**		**0.13**
	36	**0.12**	**0.12**	**0.12**		**0.13**		**0.12**		**0.12**
	60	0.12	0.12	**0.12**		**0.12**		0.12		0.12
Mean–variance	CA	0.12	0.12	**0.12**		**0.14**		**0.13**		**0.16**
	12	**0.12**	**0.14**	**0.12**		**0.16**		**0.13**		**0.20**
	36	**0.12**	**0.13**	**0.12**		**0.15**		**0.12**		**0.19**
	60	**0.12**	**0.12**	**0.12**		**0.14**		**0.11**		**0.17**
Bayes–Stein	CA	0.12	0.11	**0.12**		**0.12**		**0.12**		**0.13**
	12	**0.12**	**0.13**	**0.12**		**0.15**		**0.13**		**0.18**
	36	0.12	0.12	**0.12**		**0.13**		**0.11**		**0.15**
	60	0.12	0.12	**0.11**		**0.12**		**0.11**		**0.13**

This table reports the annualized returns and annualized volatilities for each constructed portfolio. The results cover the full period between May 2007 and November 2020. The bold marked pairs indicate the cases in which the factor optimization achieved better results

Widening the level of restrictions (columns 3 to 6) substantially increases the volatility of the sector portfolios, whereas the volatility of the factor portfolios remains at low levels. Accordingly, the largest risk differences occur among the least constrained portfolios with short and long positions between −50% and 50% (column 5 and 6 in Table 5 Panel B), respectively.

Comparing the mean returns presented in Table 5 Panel A suggests quite similar results relative to the Sharpe ratio and the risk results. In all reported cases, the factor-based portfolios yield larger or equal mean returns compared to the sector-based portfolios. The relationship between the returns and the allocation constraints is similar to the one we observed when assessing the Sharpe ratio differences for both strategies. Extending the level of the allocation freedom increases the return differences between factor and sector portfolios.

(d) Multifactor performance analysis: Fama–French factors (Table 6)

**Table 6:** Multifactor alphas and differences in alphas—Fama–French factors

Optimization method	Estimation window	Weight constraints						
		[0%; +35%]			[−35%; +35%]		[50%; +50%]	
		(1) FI	(2) SI		(3) FI	(4) SI	(5) FI	(6) SI
*Panel A: Multifactor alphas—Fama–French factors*								
1/N		0.0128***	0.0122***					
Risk parity		0.0074***	0.0075***					
MinimumVar.		0.0052***	0.0047***		0.0039***	0.0040***	0.0038***	0.0040***
Black–Litterman	CA	0.0104***	0.0090***		0.0105***	0.0092***	0.0104***	0.0092***
	12	0.0104***	0.0073**		0.0103***	0.0065**	0.0106***	0.0064***
	36	0.0115***	0.0096***		0.0116***	0.0097***	0.0119***	0.0097***
	60	0.0120***	0.0104***		0.0120***	0.0103***	0.0122***	0.0104***
Mean–variance	CA	0.0072***	0.0049**		0.0065***	0.0020	0.0071***	0.0011
	12	0.0097***	0.0070**		0.0094***	0.0015	0.0104***	0.0005
	36	0.0099***	0.0091***		0.0096***	0.0050	0.0092***	0.0047
	60	0.0100***	0.0086***		0.0098***	0.0066*	0.0096***	0.0079*
Bayes–Stein	CA	0.0052***	0.0042***		0.0050***	0.0024*	0.0051***	0.0023
	12	0.0088***	0.0064**		0.0090***	0.0006	0.0093***	− 0.0013
	36	0.0087***	0.0065***		0.0077***	0.0047	0.0075***	0.0035
	60	0.0099***	0.0071***		0.0091***	0.0074	0.0096***	0.0076**
*Panel B: Differences in alphas—Fama–French factors*								
1/N Risk parity MinimumVar.		0.0006						
		0.0007						
		0.0004			− 0.0001		− 0.0002	
Black–Litterman	CA	0.0015			0.0012		0.0012	
	12	0.0031			0.0039		0.0043	
	36	0.0019			0.0019		0.0021	
	60	0.0015			0.0017		0.0018	
Mean–variance	CA	0.0023			0.0046		0.0061*	
	12	0.0027			0.0080		0.0100	
	36	0.0008			0.0046		0.0045	
	60	0.0014			0.0032		0.0017	
Bayes–Stein	CA	0.0010			0.0026		0.0028	
	12	0.0024			0.0083 *		0.0106*	
	36	0.0021			0.0030		0.0040	
	60	0.0028			0.0017		0.0020	

Panel A of this table provides the annualized alphas of the factor and sector portfolios (FI, SI). The evaluation period covers the entire sample period from May 2007 to November 2020. *, ** and *** indicate the significance of the hypothesis test that the values displayed are different from zero at the 10%, 5% and 1% significance leve

Panel B displays the differences in alpha and the equivalent significance levels of the hypothesis test: ${H}_{0}: {Coefficient}_{FI}-{Coefficient}_{SI}=0$

Next, we next analyze the performance of the optimized portfolios within a multifactor model framework. We regress the returns of our portfolios on the six Fama–French US equity long–short factors: “Market,” Value (“HML”), Size (“SMB”), Quality (“RMW”), Investment (“CMA”) and Momentum (“MOM”) augmented by the betting-against-beta factor (“BAB,” Frazzini and Pedersen 2014). Table 6 Panel A depicts the multifactor alphas of the factor and sector portfolios along with the significance levels for the null hypothesis that alphas are different from zero. Table 6 Panel B contains the differences between alphas of factor and sector portfolios.

In Table 6, we observe positive multifactor alphas for all optimized portfolios. For the long-only case, all portfolios have positive alphas with the vast majority being significantly larger than zero. More specifically, all factor portfolios have statistically significant positive multifactor alphas, while for sector portfolios many alphas are not significantly larger than zero. Moreover, for all risk–return optimization models we find that alphas are larger for factor portfolios compared to sector allocations. In contrast, for risk-based allocations (risk parity and minimum variance) factor portfolios reveal lower multifactor alphas than sector portfolios.

This finding is in line with our earlier evidence that sectors are less correlated than factors. It also supports the conclusion of Briere and Szafarz (2021) that sector investing helps to reduce risks during crisis periods, while factor investing can boost returns during expansion periods. The highest alpha (1.22% per month) we obtain with the factor-based Black–Litterman optimization method with 60-month estimation window and with short sales allowed (weights between −50% and 50%). In line with Bessler et al. (2017), we find the largest multifactor alphas for BL portfolios compared to all other allocation strategies. Overall, the multifactor analysis confirms our finding that factor portfolios dominate sector portfolios at least for risk–return optimization models.

(e) Multifactor performance analysis: difference between both strategies

In Table 6 Panel B, we provide additional insights into the performance difference between sector and factor portfolios. In contrast to the results in Table 6 Panel A, where we reported the significance levels for the null hypothesis that alphas are different from zero, we now test for the differences in the portfolio’s factor exposures using dummy regressions. Panel B provides the difference between the alpha quotients of both allocation strategies. The positive alpha differences suggest that factor portfolios have larger multifactor alphas than sector portfolios. Comparing the three columns in Panel B, we observe that the alpha differences between the sector and factor strategies tend to become larger with wider investment restrictions, illustrating that the advantages of factor portfolios increase when relaxing investment constraints. However, due to our relatively short evaluation period, the differences in multifactor alphas between factor and sector portfolios are mostly statistically insignificant, yet economically relevant.

(f) Multifactor performance analysis: MSCI factors (Table 7)

**Table 7:** Multifactor alphas—MSCI factors

Optimization method	Estimation window	Weight constraints							
		[0%; +35%]			[−35%; +35%]		[50%; +50%]		
		(1) FI	(2) SI		(3) FI	(4) SI	(5) FI		(6) SI
1/N		0.0083*	0.0438**						
Risk parity		0.0073**	0.0461**						
MinimumVar		0.0009*	0.0047**		0.0028	0.0514**	− 0.0064		0.0518**
Black–Litterman	CA	0.0028	0.0413**		0.0013	0.0369**	− 0.0006		0.0384**
	12	0.0112**	0.0327*		0.0113*	0.0192	0.0143*		0.0152
	36	0.0064*	0.0345*		0.007	0.024	0.0076		0.0257
	60	0.0032	0.0432**		0.0017	0.0349*	0.0024		0.0367**
Mean–variance	CA	0.0069*	0.0256		0.0018	0.0247	− 0.0045		− 0.0046
	12	0.0075*	0.0162		0.0082	0.0254	0.0073		0.0071
	36	0.0014	0.0221		− 0.0039	− 0.0079	− 0.0105		− 0.0307
	60	0.0078**	0.0326*		0.006	0.0422*	0.0104		0.0321
Bayes–Stein	CA	0.0015	0.0354*		− 0.0029	0.0323	− 0.0083		0.0203
	12	0.0065*	0.0157		0.0087	0.0261	0.0062		0.005
	36	0.0028	0.0281*		− 0.0067	0.0222	− 0.0102		0.014
	60	0.0041	0.0394**		0.0023	0.0474*	− 0.001		0.0405*

This table provides the annualized alphas of the factor and sector portfolios (FI, SI). The evaluation period covers the entire sample period from May 2007 to November 2020. As return forecasts for the optimization models, we apply different moving averages, the length of which is displayed in the second column of the table. *, ** and *** indicate the significance of the hypothesis test that the values displayed are different from zero at the 10%, 5% and 1% significance level.

Next, we assess the potential outperformance of a dynamic factor timing strategy compared to a buy-and-hold factor portfolio. For this, we compute another set of multifactor alphas by regressing the returns of the optimized factor portfolios on its own components (the single MSCI factors). If the optimized portfolio was a buy-and-hold portfolio of any initial weighting, the estimated alpha would be zero and the underlying assets would fully explain the portfolio where the regression coefficients would equal the respective factor weights. In this case, the more passive the management of the underlying constituents, the closer to zero is the estimated alpha. In contrast, since the portfolios consist entirely of the same factors applied in the multifactor regression, the source of the alpha in the factor portfolios stems uniquely from the dynamic factor allocation.

While the effect of the “right bet at the right time’ might still be essential in the case of the industry allocation, here, the reported alphas contain another effect: the performance contribution of the diversification of the single sector component as well as the diversification from combining different sectors in a global portfolio. In summary, the comparison of the factor and sector alphas covers the difference between the two risk narratives described in Section 1.

In Table 7, we present the results (alpha) from the multifactor performance analysis. For most of the optimized portfolios, we observe positive multifactor alphas. For the long-only portfolios, all portfolios show positive alphas with the vast majority being significantly larger than zero. This supports our conjecture that combining the factors in an optimized portfolio results in higher returns than the return of a static buy-and-hold factor portfolio. Simply combining factors in an equally weighted portfolio (1/N) with monthly rebalancing back to the 1/N weights already provides a low but statistically significant outperformance compared to a buy-and-hold portfolio. Again, the factor-based Black–Litterman optimization method with short sales allowed (12-month estimation window and weights between −50% and 50%) provides the highest alpha (1.43% per month).

In contrast to the Fama–French factor analysis in section e), we find sector portfolios yielding higher multifactor alphas. This might appear surprising, as it seems to contradict our earlier results. However, it is very reasonable to expect that the same underlying factors explain very well factor portfolios that consist of these factors. Particularly, factors can better explain returns of factor-optimized portfolios, based on the same underlying factors, rather than portfolios consisting of sectors.

(g) Portfolio turnover (Table 8)

**Table 8:** Portfolio turnover and maximum drawdown

Optimization method	Estimation window	Weight constraints					
		[0%; +35%]		[−35%; +35%]		[−50%; +50%]	
		(1)	(2)	(3)	(4)	(5)	(6)
		FI	SI	FI	SI	FI	SI
Panel A: Portfolio turnover							
	1/N	**0.11**	0**.**27	**0.11**	0**.**27	**0.11**	0**.**27
	RP	**0.27**	0**.**51	**0.27**	0**.**51	**0.27**	0**.**51
	Min Var	**0.88**	1**.**76	**1.84**	4**.**10	**2.94**	5**.**18
BL	CA	**1.11**	1**.**22	**1.38**	1**.**69	**1.71**	1**.**71
	12	**3.01**	3**.**97	**4.29**	5**.**61	**5.60**	6**.**16
	36	**1.68**	2**.**15	**2.56**	3**.**12	**3.62**	3**.**37
	60	**1.44**	1**.**88	**2.13**	2**.**70	3**.**34	2**.**77
MV	CA	**1.28**	1**.**31	**0.85**	2**.**24	**1.51**	4**.**02
	12	**3.94**	4**.**93	**6.12**	11**.**68	**9.76**	16**.**97
	36	**2.16**	3**.**36	**3.29**	7**.**74	**5.04**	11**.**82
	60	**1.32**	2**.**73	**3.02**	5**.**52	**3.69**	8**.**81
BS	CA	**1.00**	1**.**76	**1.66**	4**.**06	**2.29**	5**.**30
	12	**3.45**	4**.**18	**5.54**	10**.**63	**8.99**	16**.**00
	36	**1.71**	2**.**67	**3.37**	5**.**93	**4.81**	8**.**40
	60	**1.05**	2**.**02	**2.40**	4**.**61	**3.28**	6,50
Panel B: Maximum drawdown							
	1/N	**0.20**	**0.21**				
	RP	**0.20**	**0.21**				
	Min Var	0.19	0.16	0.19	0.10	0.20	0.10
BL	CA	**0.20**	**0.20**	**0.20**	**0.20**	**0.20**	**0.20**
	12	0.20	0.20	0.20	0.20	**0.20**	**0.20**
	36	**0.20**	**0.20**	**0.20**	**0.20**	**0.20**	**0.20**
	60	**0.20**	**0.20**	**0.20**	**0.20**	**0.20**	**0.20**
MV	CA	0.19	0.17	0.20	0.15	**0.22**	**0.26**
	12	**0.17**	**0.19**	**0.19**	**0.22**	**0.17**	**0.30**
	36	0.17	0.17	0.15	0.15	**0.14**	**0.26**
	60	**0.18**	**0.19**	0.15	0.12	**0.11**	**0.26**
BS	CA	0.19	0.16	0.20	0.14	0.20	0.14
	12	**0.17**	**0.18**	**0.18**	**0.20**	**0.17**	**0.25**
	36	0.18	0.16	0.16	0.14	**0.14**	**0.14**
	60	0.19	0.16	0.15	0.11	0.13	0.12

This table reports the yearly portfolio turnover and the maximum drawdown for each constructed portfolio. The results cover the full portfolio period between May 2007 and November 2020. The bold marked pairs indicate the cases in which the factor optimization achieved better results.

Next, we analyze portfolio turnover of factor and sector allocations. Higher portfolio turnover is associated with larger transaction costs. Our analyses so far included 20 basis points of transaction costs, which is reasonable, given that we implement the sector and factor allocations with exchange-traded funds (ETFs). In this section, we analyze whether our assumption on transaction costs affects our results. Table 8 Panel A presents the portfolio turnover for all factor and sector portfolio. We find that for almost all allocation strategies, portfolio turnover is lower for factor compared to sector portfolio. Therefore, the relative advantage of factor compared to sector allocations increases with larger transaction costs.

### Risk analysis

An important aspect of the performance analysis is an in-depth risk analysis. For this, we analyze whether the performance difference between factor and sector portfolios is attributable to different levels of tail risk, such as maximum drawdown, or to the skewness and kurtosis of the return distribution.

- Maximum drawdown

The first part of the risk analysis covers the comparison of the maximum drawdowns (MDD) that occurred during the full investment period in factor and sector portfolios. The maximum drawdown represents the absolute losses between the highest peak and the subsequent lowest trough of the portfolio. Table 8 Panel B presents the maximum drawdowns for all strategies over the full evaluation period. Analyzing the results, we do not find a strong relationship between the MDD and the portfolio allocation strategy. The factor-based allocations have even lower drawdowns in most of the analyzed cases. For 1/N and the risk-based allocation frameworks (RP and MinVar), MDD is lower for sector portfolios than for factor portfolios. In contrast, for the risk–return optimization strategies (BL, MV, BS) MDD is lower for factor portfolios than for sector portfolios in the majority of the cases.

(b) Skewness analysis

Next, we analyze the skewness of portfolio returns. In general, investors seek positive skewness since it translates in a higher likelihood of positive returns or positive outliers. The negative skewness, in contrast, represents in general the higher likelihood of occurrences on the negative side of the return distribution (losses). For brevity, the results for the skewness analysis is available in the online appendix. Our results suggest that all of our optimized portfolios reveal negative skewness, meaning that tail risks on the negative side (left) is higher than normally distributed returns. In general, there is no clear relationship between skewness and the allocation framework. On the one hand, for 1/N and the risk-based allocation frameworks (RP and MinVar), portfolio skewness is higher for sector portfolios than for factor portfolios. On the other hand, for the risk–return optimization strategies (BL, MV, BS), the skewness is higher for factor portfolios than for sector portfolios in the vast majority of the cases. Overall, a higher skew risk cannot explain the higher returns of the factor portfolio as the skewness is even lower for most factor portfolios.

(c) Kurtosis analysis

Kurtosis characterizes the second key property of the return distributions. In general, kurtosis represents the “tailedness” of the respected distributions. Our results for the kurtosis analysis are available on the online appendix. In general, we find that all observed portfolios show leptokurtic distributions, meaning that the extreme return observations occur more frequently than expected for normally distributed returns. Similar to the skewness analysis, the results do not reveal a clear pattern how the skewness relates to factors or sectors. However, on average, and in most analyzed cases, the sector portfolios have a slightly lower excess kurtosis than factor portfolios. However, as the excess kurtosis captures both, extreme positive and negative returns, the higher kurtosis of most factor portfolios might be due to periods of high factor returns.

Our conclusion from the MDD and the overall risk analysis is that the higher returns and Sharpe ratios of the factor portfolios are not explainable with the higher tail risks of these portfolios. Moreover, we do not find a clear difference between factor and sector portfolios with regard to tail risk measures.

### Sub-period analysis

Following the analysis of the full period, we now investigate the performance for different optimization strategies and for different sub-periods. For this, we split the time series of the optimized portfolios in several sub-periods based on the state of the economy, where we distinguish between two different states: economic expansion and economic recession. As an indicator for the different states of the economy, we use the NBER recession dummy published by the US National Bureau of Economic Research.

Based on NBER recession dummies, we divide the full sample into three sub-periods. The first sub-period spans the period from May 2007 to July 2009 and includes the global financial crisis. The second sub-period contains the subsequent recovery of the global economy and financial markets. The last sub-period begins in 03/2020 due to the Corona crisis. The results of the sub-period analysis, expressed as the difference between the Sharpe ratios of the factor- and the sector-based portfolios, we present in Table 9. We structure the results again in the same way as we did for the Sharpe ratios in Table 4. In each Panel of Table 9, we present the results for one restriction, moving from strictest to moderate to lowest restrictions.

**Table 9:** Sub-periods according to the NBER indicator

		Black–Litterman			Mean–variance			Bayes–Stein		
		5/2007–7/2009	8/2009–2/2020	3/2020–11/2020	5/2007–7/2009	8/2009–2/2020	3/2020–11/2020	5/2007–7/2009	8/2009–2/2020	3/2020–11/2020
*Panel A: Sub-period analysis for the long-only portfolios with weight restriction between 0% and +35% (Sharpe ratio differences)*										
1/N	–	*− 0.54*	0.47	*− 0.41*						
RP	–	*− 0.62*	0.34	*− 0.43*						
Min Var	–	*− 0.12*	0.28	*− 0.33*						
Optimized	CA	*− 0.32*	0.36	*− 0.53*	*− 0.19*	0.73	*− 0.22*	*− 0.23*	0.41	*− 0.29*
	12	*− 0.07*	0.50	*− 0.44*	0.09	0.54	*− 0.13*	0.07	0.44	*0.01*
	36	*− 0.13*	0.43	*− 0.50*	0.18	0.43	*− 0.28*	0.03	0.39	*− 0.37*
	60	*− 0.26*	0.31	*− 0.51*	*− 0.08*	0.40	*− 0.19*	*− 0.11*	0.31	*− 0.37*
*Panel B: Sub-period analysis for the long-only portfolios with weight restriction between − 35% and +35% (Sharpe ratio differences)*										
1/N	–	*− 0.54*	0.47	*− 0.41*						
RP	–	*− 0.62*	0.34	*− 0.43*						
Min Var	–	0.83	0.20	*− 1.21*						
Optimized	CA	0.09	0.34	*− 0.66*	1.13	0.88	*− 1.24*	1.13	0.37	*− 1.31*
	12	1.53	0.54	*− 0.74*	0.64	0.65	*− 1.62*	0.60	0.57	*− 1.42*
	36	0.63	0.45	*− 0.64*	1.87	0.62	*− 0.87*	1.51	0.29	*− 1.23*
	60	0.17	0.32	*− 0.57*	2.25	0.32	*− 0.99*	1.28	0.16	*− 1.59*
*Panel C: Sub-period analysis for the long-only portfolios with weight restriction between −50% and +50% (Sharpe ratio differences)*										
1/N	–	*− 0.54*	0.47	*− 0.41*						
RP	–	*− 0.62*	0.34	*− 0.43*						
Min Var	–	0.83	0.20	*− 1.21*						
Optimized	CA	0.09	0.34	*− 0.66*	1.13	0.88	*− 1.24*	1.13	0.37	*− 1.31*
	12	1.53	0.54	*− 0.74*	0.64	0.65	*− 1.62*	0.60	0.57	*− 1.42*
	36	0.63	0.45	*− 0.64*	1.87	0.62	*− 0.87*	1.51	0.29	*− 1.23*
	60	0.17	0.32	*− 0.57*	2.25	0.32	*− 0.99*	1.28	0.16	*− 1.59*

This table reports the Sharpe ratio differences between the factor and sector portfolios. The italics values denote negative values (i.e., ${SR}_{FI}<{SR}_{SI}$). We define sub-periods by splitting the full period sample based on the NBER recession dummy, published by the National Bureau of Economic Research.

- Strictest restrictions

In Table 9 Panel A, we present the results for the portfolios with portfolio weight restrictions between 0% and +35%. The results suggest that this separates the dominance of one over the other strategy into two groups: During both crisis periods, the global financial crisis and the Covid-19 crisis, sector portfolios outperformed factors portfolios, indicating that sector portfolios offer higher diversification potential particularly during crisis periods relative to factor portfolios. This finding is in line with Briere and Szafarz (2021) who report that sector investing helps to reduce risks during crisis periods, while factor investing can boost returns during expansion periods. Consequently, during the long expansion period from August 2009 to February 2020, factor allocation clearly outperformed sector allocations. While the sector allocations dominate in two out of three sub-periods, it is important to recognize that the two sub-periods in which sectors outperform are relatively short, spanning only 11 and 9 months. In contrast, the second sub-period in which factor portfolios dominate stretches over more than 10 years. Therefore, for the full period we find factor portfolios clearly outperforming sector portfolios.

(b) Moderate restrictions

The results for the moderately constrained portfolios (restrictions between −35% and +35%) we report in Table 9 Panel B. Compared with the long-only case (Panel A of Table 9), we find similar results for the second and third sub-periods with factor portfolios dominating during the long second sub-period and sector portfolios outperforming during the short Covid-19 crisis period. However, for the first sub-period (Global Financial Crisis), relaxing the weight restrictions inverses the results. While for long-only portfolios, sector portfolios dominated during the Global Financial Crisis, for portfolios with short positions, the factor portfolios also dominate during this period.

(c) Lowest restrictions

The results for portfolios with the lowest restrictions we report in Table 9 Panel C. In general, the structure of the performance differences remains the same as for the moderate restrictions (Panel B) with only minor differences. As in the case with moderate restrictions, factor portfolios dominate in the first and second sub-period. Only in the third sub-period (Covid-19 crisis), sector allocations achieved larger Sharpe ratios.

One conclusion from the sub-period analyses is the observation that the performance differences between the sector- and factor-based allocations are not stable over time. The relative performance of sector and factor portfolios seems to relate to the state of the economy. While overall and for the full period factor portfolios dominate sector portfolios, the latter seem to be beneficial during crisis periods. However, the available history of factor indices is still too short to allow robust conclusions. Adjusting the relative size of short and long positions, optimization algorithms and different estimation window length in the optimization process, affects the comparative results, but do not fundamentally change the pattern of the relative performance.

## Conclusions

In this study, we compare the performance of two different low-cost asset allocation strategies, one building on investable factors and the other one on investable sectors, both via ETFs. We extent the earlier research of Briere and Szafarz (2021) in different directions. While Briere and Szafarz (2021) build on Fama–French factors that are not directly investable, we focus on investable factor and sector indices and in addition analyze a variety of different out-of-sample investment strategies.

For the entire investment period between May 2007 and November 2020, we find that factor portfolios provide a superior performance relative to sector portfolios. Even though the differences are not always statistically significant, which might be due to the relatively short evaluation period, the results are economically relevant with substantial Sharpe ratio differences. The results are consistent among all analyzed asset allocation strategies and estimation window length for the input parameters. The reason for the results become more evident when we analyze the performance differences in more detail. The factor portfolios generate higher average returns with lower risk (volatility). Our analysis of tail risk measures such as skewness or kurtosis of returns or the maximum drawdown reveals that factor portfolios do not exhibit larger levels of tail risk. Therefore, higher risk cannot explain the superior performance of factor portfolios. Moreover, we find that for almost all allocation strategies, portfolio turnover is lower for factor compared to sector portfolios. Hence, for larger transaction costs the relative benefits of factor compared to sector allocations become even more pronounced.

Based on multifactor regressions, we observe that both factor and sector allocations yield positive multifactor alphas when including the six Fama–French long–short factors (“Market,” Value (“HML”), Size (“SMB”), Quality (“RMW”), Investment (“CMA”) and Momentum (“MOM”)) augmented by the betting-against-beta (BAB) factor (Frazzini and Pedersen 2014). Consistent with the Sharpe ratio results, we find for all risk–return optimization models that factor portfolios provide higher multifactor alphas compared to sector portfolios. Only for risk-based allocations, sector portfolios partially provide larger multifactor alphas than factor portfolios. This result is in line with our finding that sectors reveal a lower correlation structure and hence a higher diversification potential than long-only factors.

To analyze the potential benefits of factor timing, we regress factor portfolios on the same six MSCI long-only factors, which we employ as assets in the factor portfolios. We find that risk–return optimization as well as risk-based allocations add value compared to a buy-and-hold factor strategy. Finally, our sub-period analysis indicates that during “normal” times factor portfolios clearly dominate sector portfolios, whereas during crisis periods sector portfolios are superior offering better diversification opportunities. This finding is in line with Briere and Szafarz (2021) who reported that sector investing reduces risks during crisis periods, while factor investing can boost returns during expansion periods.

However, for the full sample, we find a clear outperformance of factor portfolios. Overall, factor indices offer an attractive investment universe and are already investable for instance via ETFs. For further research, it might be interesting to investigate whether combining sectors and factors in a single portfolio adds additional value. Briere and Szafarz (2020) propose blended portfolios, which combine the diversification benefits of sector investing particularly during crisis periods with the risk premiums of factor investing. Analyzing the benefits of blended portfolios, we leave for further research.

## Supplementary Information

Below is the link to the electronic supplementary material.

Supplementary file1 (DOCX 15 kb)

## Footnotes

1 Kim et al. (2017) provide an extensive literature review on the topic of factor allocation. They conclude that despite the ambiguity between factors and assets, incorporating the factor models in the investment process might improve the investment strategy.

2 Ang et al. (2009) and Ang (2014) support the idea of factors representing different risk classes and, therefore, of independent return sources.

3 Critical views on the factor allocations approach have also been stated by Asness (2016), Asness et al. (2017) as well as Lee (2017)

4 See Anderson et al. (2012) or Maillard et al. (2010), for a discussion of this class of models.

5 Coqueret (2015) summarizes the most important reasons for the higher attention. First, in contrast to the “classical” optimization methods, no return forecasting is necessary, which could have considerable effects on the allocation outcome (Kondor et al. 2007). Second, in crisis-periods investors tend to buy lower-risk products. Finally, since Black (1972) as well as Haugen and Heins (1972) introduced the so-called low-volatility paradox, a large body of literature concludes that lower-risk assets do not necessarily perform worse than their higher-risk counterparts do.

6 Mean–variance relies on the critical assumption that asset returns are normally distributed. Although this requirement is often identified as one of the main weaknesses in academia, Landsman and Nešlehová (2008) demonstrate that it is sufficient that returns are elliptically symmetrically distributed so that all investor preferences are equivalent to mean–variance preferences. Therefore, it is sensible to apply the mean–variance framework for portfolio optimization even if asset returns are non-normal, as long as they are symmetric.

7 For a detailed exposition see Bessler et al. (2017) and Bessler and Wolff (2015).

8 For an application, see Bessler et al. (2017, 2021), Bessler and Wolff (2015). In the sample-based version of the BL model “views” are the sample means of the respective asset returns. The reliability of “views” is measured as the variance of the historical forecast errors ε_i_ during the sample period “Implied” returns are computed based on the 1/N portfolio. The parameter τ is set to 0.05. Earlier studies use similar values ranging from 0.025 to 0.3 (Black and Litterman 1992; He and Litterman 2002; Idzorek 2005).
