# Supplementary material for: Factor investing and asset allocation strategies: a comparison of factor versus sector optimization
Source: J Asset Manag. 2021 May 29;22(6):488–506. doi: 10.1057/s41260-021-00225-1 (PMC8164058; doi:10.1057/s41260-021-00225-1)
Supplement: Supplementary file 1 — Supplementary file1 (DOCX 15 kb) [file 41260_2021_225_MOESM1_ESM.docx]

Online Appendix:

Table 10. Tail risk measures

|  |  | Weight Constraints | | | | | | | | |
| --- | --- | --- | --- | --- | --- | --- | --- | --- | --- | --- |
|  |  | [0%; +35%] | | [-35%; +35%] | | | | [-50%; +50%] | | |
|  |  | (1) (2) | | (3) (4) | | | | (5) (6) | | |
| Optimization  Method | Forecast  Length | FI | SI | | FI | SI | FI | | SI |  |
| Panel A: Skewness of portfolio returns | | | | | | | | |  |  |
|  | 1/N | -0.76 | -0.58 | |  |  |  | |  |  |
|  | RP | -0.80 | -0.67 | |  |  |  | |  |  |
|  | Min Var | -0.80 | -0.78 | | -0.79 | -0.84 | -0.82 | | -0.65 |  |
| BL | CA | -0.86 | -0.78 | | -0.86 | -0.83 | -0.84 | | -0.75 |  |
|  | 12 | **-0.85** | **-0.91** | | **-0.87** | **-0.96** | **-0.84** | | **-1.07** |  |
|  | 36 | **-0.85** | **-0.87** | | **-0.88** | **-0.99** | **-0.85** | | **-0.90** |  |
|  | 60 | -0.85 | -0.84 | | **-0.85** | **-0.88** | **-0.82** | | **-0.82** |  |
| MV | CA | -0.81 | -0.62 | | -0.79 | -0.69 | -1.04 | | -0.79 |  |
|  | 12 | **-0.58** | **-0.69** | | -0.75 | -0.41 | -0.49 | | -0.42 |  |
|  | 36 | **-0.65** | **-0.81** | | -0.64 | -0.60 | **-0.42** | | **-0.68** |  |
|  | 60 | **-0.64** | **-0.89** | | **-0.54** | **-0.57** | **-0.30** | | **-0.71** |  |
| BS | CA | -0.81 | -0.75 | | **-0.88** | **-0.95** | **-0.94** | | **-1.01** |  |
|  | 12 | **-0.59** | **-0.79** | | -0.67 | -0.48 | **-0.53** | | **-0.54** |  |
|  | 36 | **-0.65** | **-0.73** | | -0.63 | -0.52 | **-0.42** | | **-0.47** |  |
|  | 60 | **-0.74** | **-0.77** | | **-0.62** | **-0.73** | **-0.47** | | **-0.66** |  |
| Panel B: Kurtosis of portfolio returns | | | | | | | | | |  |
|  | 1/N | 5.10 | 4.42 | |  |  |  | |  |  |
|  | RP | 5.28 | 4.63 | |  |  |  | |  |  |
|  | Min Var | 5.08 | 4.28 | | **4.72** | **5.31** | 4.76 | | 4.19 |  |
| BL | CA | 5.24 | 4.81 | | 5.16 | 5.07 | 5.09 | | 4.79 |  |
|  | 12 | 4.89 | 4.35 | | 4.80 | 4.46 | **4.67** | | **5.04** |  |
|  | 36 | 5.06 | 4.79 | | **5.04** | **5.28** | 4.99 | | 4.83 |  |
|  | 60 | 5.09 | 4.97 | | **5.04** | **5.13** | 4.99 | | 4.91 |  |
| MV | CA | 4.92 | 3.79 | | 5.18 | 4.93 | 6.12 | | 5.43 |  |
|  | 12 | 4.06 | 3.61 | | 4.64 | 3.25 | **3.50** | | **3.53** |  |
|  | 36 | 4.32 | 3.92 | | 4.00 | 3.33 | **3.31** | | **3.82** |  |
|  | 60 | **4.37** | **4.52** | | 3.77 | 3.50 | **3.21** | | **4.22** |  |
| BS | CA | 4.98 | 4.20 | | **5.05** | **6.17** | **5.25** | | **6.52** |  |
|  | 12 | 4.10 | 3.85 | | 4.27 | 3.46 | **3.70** | | **4.03** |  |
|  | 36 | 4.49 | 3.91 | | 4.10 | 3.71 | **3.49** | | **3.58** |  |
|  | 60 | 4.82 | 4.03 | | **4.02** | **4.71** | 3.57 | | 4.64 |  |

This table reports the monthly skewness and kurtosis for each constructed portfolio. The results cover the full portfolio period between May 2007 and November 2020. The bold marked pairs indicate the cases in which the factor optimization achieved lower kurtosis.
